# A Prospective Evaluation of the Association between a Single Nucleotide Polymorphism rs3775291 in Toll-Like Receptor 3 and Breast Cancer Relapse

**DOI:** 10.1371/journal.pone.0133184

**Published:** 2015-07-30

**Authors:** Dan-Na Chen, Chuan-Gui Song, Ke-Da Yu, Yi-Zhou Jiang, Fu-Gui Ye, Zhi-Ming Shao

**Affiliations:** 1 Department of General Surgery, Affiliated Union Hospital, Fujian Medical University, Fuzhou, China; 2 Department of Breast Surgery, Key Laboratory of Breast Cancer, Fudan University Shanghai Cancer Center, Shanghai Medical College, Fudan University, Shanghai, China; Medical College of Soochow University, CHINA

## Abstract

**Background:**

Toll-like receptors (TLRs) regulate the balance between the innate and adaptive immune responses. Missense single nucleotide polymorphisms (SNPs) in *TLRs* might be functional and thus influence the risks of chronic infection and cancer development. Here, we investigated the association of two missense SNPs, rs3775291 (c.1234G>A) in the *TLR3* gene and rs4833095 (c.743T>C) in the *TLR1* gene, with relapse-free survival (RFS) in a cohort of prospectively observed breast cancer patients.

**Methods:**

In this prospective observational study, rs3775291 in *TLR3* and rs4833095 in *TLR1* were genotyped in 715 patients with primary breast cancer in a Chinese population.

**Results:**

Univariate analysis revealed that the patients with the AA genotype of rs3775291 had a shorter RFS compared with those carrying the G allele in the recessive model (P<0.01), but this finding was not observed with the dominant model (P = 0.31). The results remained significant after adjusting for the clinical parameters in the recessive model (HR = 3.53, 95% confidence interval [CI]: 1.98–6.31, P<0.01). Further survival analysis indicated that this SNP was significant in the luminal-B, triple-negative breast cancer (TNBC), and human epidermal growth factor receptor 2-positive (HER2+) patients using the recessive model but that it was not significant in the luminal-A patients. The SNP rs4833095 showed a non-significant tendency toward an increased RFS rate in the patients with the TT genotype.

**Conclusion:**

Our results suggest that the SNP rs3775291 in *TLR3* may influence patient outcome. Further studies with larger sample sizes should be conducted to validate our findings.

## Introduction

Breast cancer is the most frequently diagnosed type of cancer [1], and the risk of recurrence is influenced by the stage at initial presentation and the underlying biology of the tumor. Despite the known prognostic factors for breast cancer, including tumor size, nodal involvement, grade, lymphovascular invasion, and the estrogen receptor (ER) and human epidermal growth factor receptor 2 (HER2) statuses [2], relapse is still difficult to predict.

Several studies have suggested that imbalances between inflammatory- and immune-associated proteins contribute to breast cancer and disease progression [3]. Persistent inflammatory conditions stimulate the production of cytokines and chemokines, which promote angiogenesis, metastasis, and subversion of adaptive immunity [4]. Toll-like receptors (TLRs) play important roles in the innate and adaptive immune responses. They selectively recognize a variety of conserved molecular structures in invading pathogens, initiating complex downstream signaling pathways, including the NF-κB and MAPK pathways, ultimately resulting in a cytokine profile that is associated with immune tolerance and cancer progression [5,6,7]. In the TLR family, TLR3 is found on the surfaces of endosomes and mainly binds to double-stranded RNA (dsRNA), whereas TLR1 is found on outer membranes and recognizes various bacterial components [8]. Several lines of evidence have indicated that TLR3 plays important roles in breast cancer development and progression. For example, high TLR3 expression in breast tumor cells has been found to be related to tumor aggressiveness and metastasis [9]. In addition, treatment with double-stranded RNA (dsRNA) has been shown to be associated with a significant decrease in the risk of relapse in TLR3-positive breast cancer [10].

Missense single nucleotide polymorphisms (SNPs) within *TLR* genes can affect TLR functionality and potentially alter the balance between the pro- and anti-inflammatory responses and influence the risks of chronic infection and cancer development [11]. Specifically, the SNP rs3775291 is one of the most important SNPs in the *TLR3* gene. A few studies have reported the association between SNPs in *TLR3* and various types of cancer, such as oral cancer [12], colorectal cancer and lung cancer [13,14]. Interestingly, all of these studies have identified rs3775291 and have suggested that the AA genotype is associated with increased risk. In addition, rs3775291 has been reported to play roles in some infectious diseases and has been shown to cause a missense mutation at amino acid (aa) 412, altering leucine (L) to phenylalanine (F) in the TLR3 ectodomain. Another *TLR1* gene SNP, rs4833095, is a well-studied genetic variant, and the TT genotype has been reported to be associated with increased risks of non-Hodgkin lymphoma (NHL) and prostate cancer [15]. This SNP leads to a missense mutation at aa 248, altering asparagine to serine (N248S), and may result in decreased receptor function. To the best of our knowledge, no studies have been conducted to evaluate the effects of rs3775291 in *TLR3* and rs4833095 in *TLR1* on breast cancer survival.

Here, we hypothesized that genetic variants in *TLR1* and *TLR3* may be associated with breast cancer outcome. To test this hypothesis, we evaluated the association of rs3775291 in *TLR3* and rs4833095 in *TLR1* with breast cancer survival in 715 primary breast cancer patients in a cohort of prospectively observed Chinese patients.

## Methods

### Ethics Statement

This study was approved by the Ethical Committee of the Shanghai Cancer Center of Fudan University, and each participant signed an informed consent document.

### Study Population

This prospective observational study was initiated in 2004. A total of 963 unrelated patients with pathologically confirmed primary breast cancer were recruited from the Shanghai Cancer Center from January 2004 to January 2007. Genotyping of rs3775291 and rs4833095 was conducted in 2008–2009 [16,17,18]. Patients selected for the present analysis fulfilled the following inclusion criteria: (i) female gender and diagnosis of unilateral invasive breast cancer; breast carcinoma *in situ* (with or without microinvasion) was excluded; (ii) pathologic examination of tumor specimens carried out at the Department of Pathology of Fudan University Shanghai Cancer Center; (iii) presence of an operable tumor, without any evidence of recurrence or metastasis at diagnosis; (iv) no receipt of neoadjuvant systemic therapy (chemotherapy and/or hormone therapy) or preoperative irradiation; (v) no previous history of other types of cancer (other than breast cancer); and (vi) availability of at least 2 months of follow-up data.

Of the 963 unrelated patients who were originally enrolled in the prospective observational study, 806 (83.70%) met the inclusion criteria and were genotyped successfully. Among these patients, 91 were excluded because complete follow-up information was not available. As a result, 715 (88.71%) patients were included in this study.The last follow-up date was October 31, 2013, and the median follow-up time was 73.4 months (ranging from 2 to 117.5 months). Clinical information was extracted from the patients’ medical records. Because the information on tumor grade was missing in many cases, we did not include this variable in our analysis. The preoperative evaluation and examination procedures used have been described elsewhere [19]. The systemic treatment strategy was updated according to the St. Gallen consensus [20,21]. The molecular subtypes of breast cancer according to immunohistochemical (IHC) profiles were categorized as follows: luminal-A = ER+ or PR+, HER2-, and Ki67 < 14%; luminal-B = ER+ or PR+ and HER2+ or Ki67 ≥ 14%; HER2-enriched (HER2+) = ER-, PR-, and HER2+; and triple-negative breast cancer (TNBC) = ER-, PR-, HER2-. Because Ki-67 was missing in some data, so the rule of classification in this part of the data was that: luminal-A = ER+ or PR+, and HER2-; luminal-B = ER+ or PR+, and HER2+; HER2-enriched (HER2+) = ER-, PR-, and HER2+; and triple-negative breast cancer (TNBC) = ER-, PR-, HER2-. The REMARK criteria of tumor marker evaluation were followed [22].

### SNP Genotyping

Genomic DNA was extracted from 3 to 5 ml of peripheral blood lymphocytes using a Gentra PureGene DNA Purification Kit (Gentra Systems, USA) according to the manufacturer’s instructions and then stored at -20°C. SNPs were genotyped with a 12-plex SNPstream Platform (Beckman Coulter Inc.) [23]. Genotyping was carried out by the Chinese National Human Genome Center (Shanghai). To confirm the genotyping results, 10% of the DNA samples were randomly selected for direct sequencing, and the results were 100% concordant.

### Statistical Analysis

Relapse-free survival (RFS) was measured from the date of diagnosis to the date of first local/regional recurrence or distant metastasis or last follow-up. Patients who died before experiencing disease recurrence were censored at their date of death in analysis. Different models were constructed to evaluate the effects of different genotypes on breast cancer survival. The dominant model was defined as major homozygotes vs. heterozygotes + minor homozygotes, the recessive model included minor homozygotes vs. heterozygotes + major homozygotes, and independent comparison between any of the two genotypes was defined as a co-dominant model. Survival curves were constructed using the Kaplan–Meier method, and they were compared by the log-rank test. Analyses of different parameters for prognostic significance, HRs for disease progression, and 95%CIs were performed using univariate and multivariate Cox proportional hazard models. Only those clinical factors with P-values of ≤ 0.10 in univariate Cox analysis were used in the multivariate Cox model. P-values of <0.05 were considered significant. SPSS version 18.0 (SPSS, Inc., Chicago, IL, USA) was used in all analyses.

## Results

### Characteristics of the Study Population

The distributions of the demographic and clinical characteristics and the genotype frequencies of rs3775291 and rs4833095 in the 715 primary breast cancer patients are presented in Table 1. During the median follow-up period, 130 patients experienced at least one site of recurrence or distant metastasis, with a 5-year RFS rate of 81.96%. The genotype frequencies of rs3775291 (AA 10.11%, AG 45.08%, and GG 44.80%) and rs4833095 (TT 14.75%, TC 47.47%, and CC 37.78%) in these patients were comparable to those previously reported in the HapMap database for the Han Chinese population (http://hapmap.ncbi.nlm.nih.gov/, HapMap Data Rel 28 Phase II+III, August 10; rs3775291: AA 7.4%, AG 37.5%, and GG 55.1%; and rs4833095: TT 11.8%, TC 41.2%, and CC 47.1%). No significant deviation from Hardy-Weinberg equilibrium was observed for the two SNPs (rs3775291: P = 0.50; and rs4833095: P = 0.94).

**Table 1:** Characteristics and genotype prevalence for 715 breast cancer patients.

Characteristics		Patients(n)	n%
Mean Age (± SD)		50.34 ± 12.05	
Age (years)			
	<50	330	46.15
	≥50	384	53.71
Menopausal Status			
	Premenopausal	383	53.57
	Postmenopausal	332	46.43
Tumor Size(cm)			
	≤2	334	46.71
	>2	290	40.56
	Unknown	91	12.73
Lymph Node Status			
	Positive	304	42.52
	Negative	355	49.65
	Unknown	56	7.83
ER Status			
	Positive	362	50.63
	Negative	175	24.48
	Unknown	178	24.90
PR Status			
	Positive	336	46.99
	Negative	201	28.11
	Unknown	178	24.90
HER2 Status			
	Positive	82	11.47
	Negative	446	62.38
	Unknown	187	26.15
Subtype			
	Luminal-A	174	24.34
	luminal-B	207	28.95
	Basal-like	99	13.85
	HER2+	53	7.41
	Unknown	182	25.45
*TLR3* rs3775291			
	AA	72	10.11
	AG	321	45.08
	GG	319	44.80
*TLR1* rs4833095			
	TT	105	14.75
	CT	338	47.47
	CC	269	37.78
Chemotherapy			
	Yes	333	46.57
	No	189	26.43
	Unknown	193	26.99
Endocrine Therapy			
	Yes	388	54.27
	No	149	20.84
	Unknown	178	24.90

ER, estrogen receptor; PR, progesterone receptor; HER2, human epidermal growth factor receptor 2

### Association between SNPs and RFS in Breast Cancer

**Fig 1:**
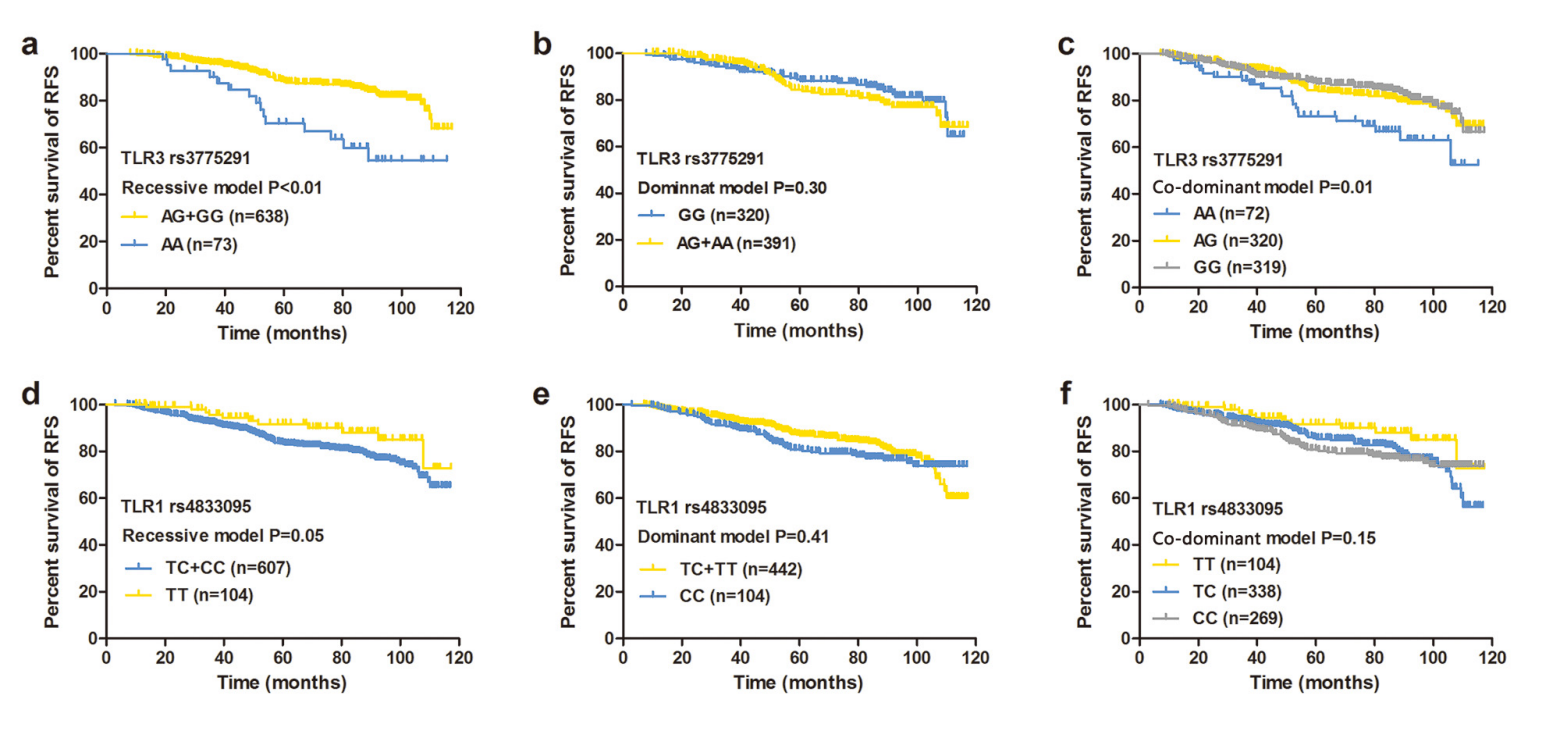
Effects of rs3775291 and rs4833095 on RFS. Kaplan-Meier estimates of RFS in 715 breast cancer patients according to the rs3775291: (a) co-dominant model, (b) dominant model, and (c) recessive model and the rs4833095: (d) co-dominant model, (e) dominant model, and (f) recessive model. P-value tested by the log-rank test.

We first analyzed the RFS rates for rs3775291 and rs4833095 in different models using the Kaplan–Meier and log-rank tests (Fig 1). Univariate analysis revealed that rs3775291 was a significant prognostic marker under the recessive and co-dominant models (recessive model: AA vs. AG+GG: HR = 2.06, 95%CI: 1.31–3.23, P<0.01; co-dominant model: AA vs. GG: HR = 2.04, 95%CI: 1.24–3.35; AG vs. GG: HR = 1.07, 95%CI: 0.73–1.56, P = 0.01; Table 2), but not under the dominant model (GG vs. AG+AA, P = 0.31). The results obtained with the recessive model remained significant in multivariate Cox regression analysis after adjusting for lymph node status, tumor size, chemotherapy, endocrine therapy, and ER, PR, and HER2 statuses (Table 3) and indicated that the patients with the AA genotype had a relatively higher risk of recurrence (HR = 3.53, 95%CI: 1.98–6.31, P<0.01) compared with those carrying the G allele. In the co-dominant model, the RFS rate for the AG genotype was almost the same as that for the GG genotype (HR = 1.01, 95%CI: 0.59–1.75, P = 0.96), and the patients with the AA genotype presented an obviously shorter RFS compared with those with the GG genotype (HR = 3.37, 95%CI: 1.74–6.51, P<0.01). Thus, the breast cancer patients with the AA genotype of the SNP rs3775291 had a worse prognosis for RFS.

**Table 2:** Univariate Cox regression analysis of RFS for different models of SNP rs3775291 and rs4833095 in 715 breast cancer patients.

Variables		HR (95%CI)	P
Age			0.60
	<50	1.00	
	≥50	0.91 (0.65–1.29)	
Menopausal status			0.87
	Premenopausal	1.00	
	Postmenopausal	0.97 (0.69–1.37)	
ER status			<0.01
	Negative	1.00	
	Positive	0.50 (0.33–0.74)	
PR status			0.01
	Negative	1.00	
	Positive	0.60 (0.40–0.88)	
HER2 status			<0.01
	Negative	1.00	
	Positive	2.13 (1.36–3.33)	
Lymph node status			<0.01
	Negative	1.00	
	Positive	2.80 (1.87–4.19)	
Tumor size(cm)			<0.01
	≤2	1.00	
	>2	2.59 (1.73–3.86)	
Chemotherapy			<0.01
	No	1.00	
	Yes	3.34 (1.92–5.82)	
Endocrine therapy			<0.01
	No	1.00	
	Yes	0.51 (0.34–0.76)	
*TLR3 rs3775291*:			
Recessive model			<0.01
	AG+GG	1.00	
	AA	2.06 (1.31–3.23)	
Dominant model			0.31
	AG+AA	1.00	
	GG	0.83 (0.59–1.18)	
Co-dominant model			0.01
	GG	1.00	
	AG	1.07 (0.73–1.56)	0.74
	AA	2.04 (1.24–3.35)	<0.01
*TLR1 rs4833095*:			
Recessive model			0.06
	TC+CC	1.00	
	TT	0.55 (0.30–1.01)	
Dominant model			0.41
	TC+TT	1.00	
	CC	1.16 (0.82–1.64)	
Co-dominant model			0.16
	CC	1.00	
	TC	0.97 (0.67–1.39)	0.85
	TT	0.54 (0.29–1.02)	0.06

ER, estrogen receptor; PR, progesterone receptor; HER2, human epidermal growth factor receptor 2

**Table 3:** Multivariate Cox regression analysis of RFS for different models of SNP rs3775291 and rs4833095.

Models		HR (95%CI)	P^a^
*rs3775291*:			
Recessive model			<0.01
	AG+GG	1.00	
	AA	3.53 (1.98–6.31)	
Dominant model			0.34
	AG+AA	1.00	
	GG	0.79 (0.48–1.29)	
Co-dominant model			<0.01
	GG	1.00	
	AG	1.01 (0.59–1.75)	0.96
	AA	3.37 (1.74–6.51)	<0.01
*rs4833095*:			
Recessive model			0.08
	TC+CC	1.00	
	TT	0.47 (0.20–1.09)	
Dominant model			0.19
	TC+TT	1.00	
	CC	1.39 (0.85–2.27)	
Co-dominant model			0.15
	CC	1.00	
	TC	0.82 (0.50–1.36)	0.44
	TT	0.41 (0.17–1.02)	0.05

ER, estrogen receptor; PR, progesterone receptor; HER2, human epidermal growth factor receptor

^a^Adjusted for lymph node status, tumor size, endocrine therapy, chemotherapy, ER, PR, and HER2 status

Kaplan–Meier analysis of rs4833095 showed a tendency toward improved survival in the patients with the TT genotype under the recessive model (P = 0.05, Fig 1), but not under the additive (P = 0.15) or dominant model (P = 0.41). However, univariate and multivariate Cox regression analyses revealed that neither of the models was significantly associated with RFS (P>0.05, Table 2). Thus, the patients with the TT genotype in SNP rs4833095 had a (non-significant) tendency toward an increased RFS rate compared with those carrying the C allele.

### Stratification Analysis of Different Molecular Subtypes of Breast Cancer

Because different molecular subtypes of breast cancer are related to unique relapse behaviors, we further analyzed the associations between the two SNPs and the RFSs for different molecular subgroups. The results of the Kaplan-Meier and log-rank tests showed that the recessive model of rs3775291 was significant for the luminal-B and TNBC patients (P<0.01 for luminal-B and P = 0.02 for TNBC), but not for the luminal-A or HER2+ patients (P = 0.54 in luminal-A and P = 0.11 in HER2+), whereas the co-dominant model was significant for the luminal-B but not for the TNBC subtypes (P<0.01 for luminal-B and P = 0.19 for TNBC, S1 Fig). However, after adjusting for clinical factors, we found that the recessive model was significant for the luminal-B (HR = 2.93, 95%CI: 1.26–6.83, P = 0.01), TNBC (TNBC: HR = 3.27, 95%CI: 1.17–9.15, P = 0.02), and HER2+ patients (HR = 12.12, 95%CI: 2.21–66.57, P<0.01, Table 4), but not for the luminal-A patients (P = 0.90).

**Table 4:** Multivariate Cox regression analysis of rs3775291 and rs4833095 in different molecular subtypes.

Models		Luminal-A (n = 174)		Luminal-B (n = 207)		TNBC (n = 99)		HER2+ (n = 53)	
*rs3775291*		HR (95%CI)	P^a^	HR (95%CI)	P^a^	HR (95%CI)	P^b^	HR (95%CI)	P^c^
Co-dominant model			0.76		0.04		0.13		0.02
	GG	1.00		1.00		1.00		1.00	
	AG	0.64 (0.19–2.18)	0.47	1.32 (0.56–3.12)	0.52	1.25 (0.45–3.48)	0.66	0.93 (0.32–2.71)	0.88
	AA	0.74 (0.09–6.27)	0.78	3.39 (1.28–8.94)	0.01	3.46 (1.01–11.87)	0.05	11.63(1.93–70.00)	<0.01
Recessive model			0.90		0.01		0.02		<0.01
	AG+GG	1.00		1.00		1.00		1.00	
	AA	0.87 (0.11–7.13)		2.93 (1.26–6.83)		3.27 (1.17–9.15)		12.12 (2.21–66.57)	
Dominant model			0.47		0.16		0.48		0.67
	AG+AA	1.00		1.00		1.00		1.00	
	GG	1.52 (0.49–4.76)		0.57 (0.26–1.26)		0.72 (0.29–1.80)		0.81 (0.30–2.16)	
*rs4833095*		HR (95%CI)	P^a^	HR (95%CI)	P^a^	HR (95%CI)	P^b^	HR (95%CI)	P^c^
Co-dominant model			0.82		0.77		0.12		0.25
	CC	1.00		1.00		1.00		1.00	
	TC	1.26 (0.39–4.01)	0.70	1.27 (0.54–3.00)	0.59	0.39 (0.16–0.96)	0.04	0.43 (0.14–1.34)	0.15
	TT	0.67 (0.08–5.81)	0.72	0.88 (0.26–2.90)	0.83	—*	0.94	0.32 (0.06–1.72)	0.18
Recessive model			0.62		0.64		0.94		0.43
	TC+CC	1.00		1.00		1.00		1.00	
	TT	0.59 (0.07–4.61)		0.77 (0.26–2.29)		—*		0.54 (0.12–2.49)	
Dominant model			0.83		0.74		0.02		0.10
	TC+TT	1.00		1.00		1.00		1.00	
	CC	0.88 (0.29–2.73)		0.87 (0.39–1.96)		3.00 (1.20–7.44)		2.49 (0.84–7.43)	

ER, estrogen receptor; PR, progesterone receptor; HER2, human epidermal growth factor receptor 2; TNBC, triple-negative breast cancer

^a^Adjusted for age, menopausal status, chemotherapy, lymph node status, and tumor size

^b^Adjusted for chemotherapy and lymph node status

^c^Adjusted for menopausal status, endocrine therapy, and lymph node status

*The values of the HR and the 95%CI were not determinable because the number of patients with TT (n = 6) was too small and no event occurred in these patients

In contrast, the SNP rs4833095 was not significantly associated with any of the four subgroups, as determined by univariate analysis (S2 Fig). Multivariate analysis showed a likely association of RFS with this SNP for the TNBC subtype (CC vs. TC+TT: HR = 3.00, 95%CI: 1.20–7.44, P = 0.02). However, the sample number of TNBC patients was too small, and the relapse events were too rare to reach a strong conclusion (Table 4).

## Discussion

Despite significant improvements in the diagnosis and treatment of breast cancer, a considerable proportion of patients experience relapse, indicating the need to discover new prognostic molecular markers for this disease. Our study aimed to explore the possible effects of the SNPs rs3775291 in *TLR3* and rs4833095 in *TLR1* on the prognosis of breast cancer patients. We performed a prospective observational study of a Chinese population, demonstrating that rs3775291 was an independent prognostic factor for RFS. In this cohort, the patients with the AA genotype had a shorter RFS than those carrying the G allele.

The exact mechanism by which rs3775291 contributes to a worse RFS remains unclear. Structural analysis of the TLR3 molecule has revealed that a glycosylation site (Asn413) within the ligand-binding surface for dsRNA is required for TLR3 activation [24]. Asn413 is located adjacent to the L412P variant, which may contribute to the alteration of ligand binding or to the dimerization of TLR3. Previous studies of some chronic inflammatory diseases have confirmed this conjecture. These studies have reported that L412F has no effects on the mRNA or protein expression level of TLR3 but that it reduces the binding capacity of TLR3 to dsRNA, thereby reducing dsRNA-induced cell death and decreasing TLR3-mediated NF-κB activation [25,26]. Therefore, we speculate that patients with the AA genotype are more likely to relapse because this variant changes the ligand-binding function of TLR3 protein, influencing the activities of the pro- and anti-inflammatory cytokines and chemokines produced following TLR3 activation, ultimately altering the antitumor- and apoptosis-inducing effects of this protein and resulting in a worse RFS.

A further stratification experiment was performed to estimate the prognostic implications of different molecular subtypes, showing that the AA genotype in rs3775291 is associated with worse RFS for the luminal-B, TNBC, and HER2+ subtypes, but not for the luminal-A subtype. In our population, we found that the frequency of the AA genotype in the luminal-A subtype (6.90%) was lower than those of the other three subtypes (luminal-B: 12.56%; TNBC: 8.08%; and HER2+: 11.32%) and that the incidence rate of events for the AA genotype in the luminal-A patients (16.67%) was also the lowest (luminal-B: 38.46%; TNBC: 50%; and HER2+: 50%). Because luminal-A breast cancer had the best prognosis, the lowest rate of local or regional relapse, and the longest median duration of survival with distant metastasis compared with the other three subtypes [2,27], we speculate that the good effect of the luminal-A subtype itself may neutralize the poor effect of the AA genotype. However, elucidating the potential underlying mechanisms requires further intensive research.

In our population, the SNP rs4833095 showed a tendency toward improved RFS in the patients with the TT genotype, although this finding was not statistically significant. However, the effect of this SNP seems limited. Although it has been reported to be associated with non-Hodgkin lymphoma and prostate cancer risks, these relationships are controversial [15,28]. Other studies have suggested that it may not be functional itself but rather may tag a causative variant [29]. In addition, TLR1, 2, 6 and 10 are closely related and comprise the TLR2 subfamily. TLR1 forms a heterodimer with TLR2. They act as a co-receptor for recognizing bacterial components and the major function depends on TLR2 [5,8]. Therefore, the change caused by a single SNP in *TLR1* may not be powerful enough to affect the binding of the TLR2-TLR1 heterodimer to its ligands and thus may not influence the downstream pathway. Furthermore, we assessed the function of rs4833095 using two types of function prediction software. This SNP was predicted to be ‘benign’ by PolyPhen-2 (version 2.2.2) (http://genetics.bwh.harvard.edu/pph2/) with a score of 0.001 and to be ‘tolerated’ by the Sorting Intolerant from Tolerant (SIFT) (http://sift.jcvi.org/) algorithm with a score of 0.18, confirming our speculation. Further analysis revealed that this SNP completely lost its effects in all subtypes except for the TNBC subtype. However, in the TNBC subgroup, the number of patients with the TT genotype (n = 6) was too small, and no events occurred in these patients. Thus, we consider the association between the TC genotype and RFS to be insignificant. Taken together, these findings suggest that rs4833095 may not affect the function of TLR1, and thus may not be associated with breast cancer RFS.

To our knowledge, this is the first study investigating the impacts of rs3775291 and rs4833095 on RFS in breast cancer. The strengths of this study include its population-based prospective design, its high rate of patient recruitment, its detailed data on established risk factors, and the relatively comprehensive follow-up information. Our study also has several limitations. First, it was conducted using a single-center design, and the number of patients included was relatively small, especially for the molecular subtypes. Second, only two SNPs were analyzed in our study. Systematic analysis of variants in TLR genes would provide a more comprehensive understanding of the effects of these SNPs on breast cancer patient outcome. Third, we need functional analyses to further investigate the exact mechanism and confirm our conjecture.

In conclusion, this study has shown for the first time that the *TLR3* SNP rs3775291 is associated with an increased risk of relapse in breast cancer, especially for the luminal B, TNBC, and HER2+ subtypes. Because a SNP is a germline variation that can be directly detected in a patient’s blood sample, it may serve as a clinical prognostic marker of malignancy. Further validation and feasibility studies are required before the results of this study can be considered for clinical use.

## Supporting Information

S1 Fig Effects of rs3775291 in different molecular subtypes. Effects of rs3775291 on RFS according to different models for Luminal-A: (a) recessive model, (b) co-dominant model; Luminal-B: (c) recessive model, (d) co-dominant model; TNBC: (e) recessive model, (f) co-dominant model; and HER2+ subtype: (g) recessive model, (h) co-dominant model. P-value tested by the log-rank test.
(TIF)

S2 Fig Effects of rs4833095 in different molecular subtypes. Effects of rs34833095 on RFS according to different models for Luminal-A: (a) dominant model, (b) co-dominant model; Luminal-B: (c) dominant model, (d) co-dominant model; TNBC: (e) dominant model, (f) co-dominant model; and HER2+ subtype: (g) dominant model, (h) co-dominant model. P-value tested by the log-rank test.
(TIF)
