# Supplementary figures and images for: A Prospective Evaluation of the Association between a Single Nucleotide Polymorphism rs3775291 in Toll-Like Receptor 3 and Breast Cancer Relapse
Source: PLoS One. 2015 Jul 30;10(7):e0133184. doi: 10.1371/journal.pone.0133184 (PMC4520586; doi:10.1371/journal.pone.0133184)

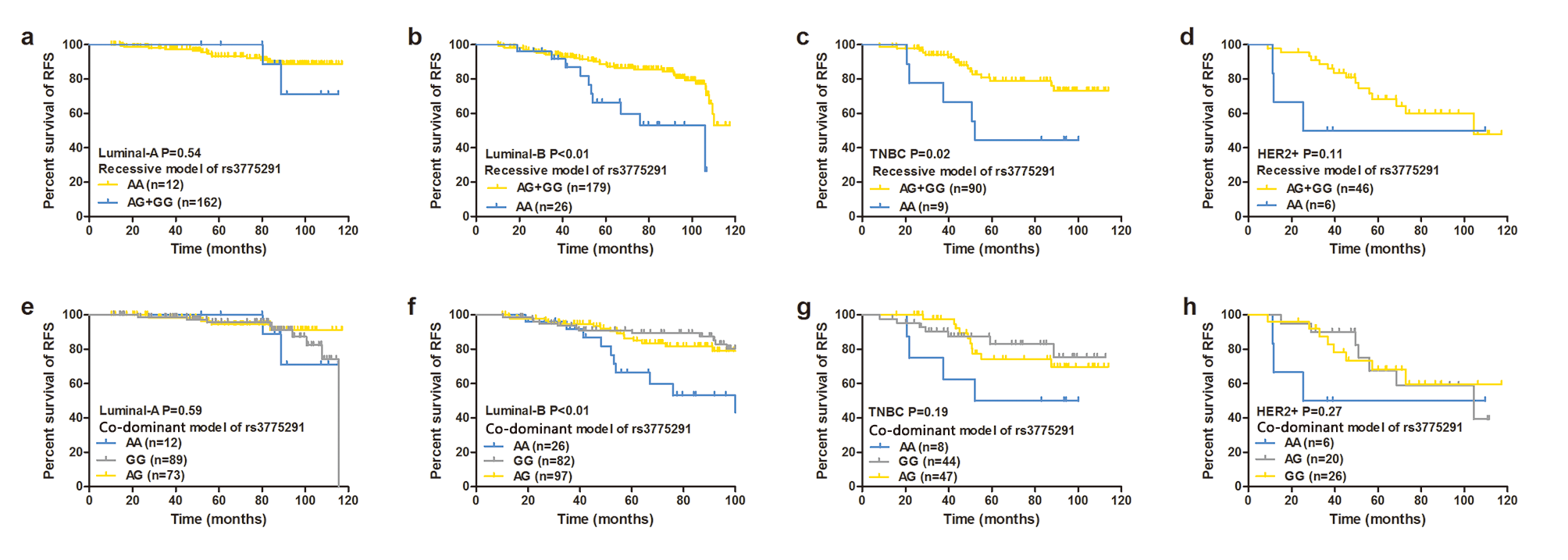

Supplement: S1 Fig — Effects of rs3775291 on RFS according to different models for Luminal-A: (a) recessive model, (b) co-dominant model; Luminal-B: (c) recessive model, (d) co-dominant model; TNBC: (e) recessive model, (f) co-dominant model; and HER2+ subtype: (g) recessive model, (h) co-dominant model. P-value tested by the log-rank test. (TIF) [file pone.0133184.s001.tif]

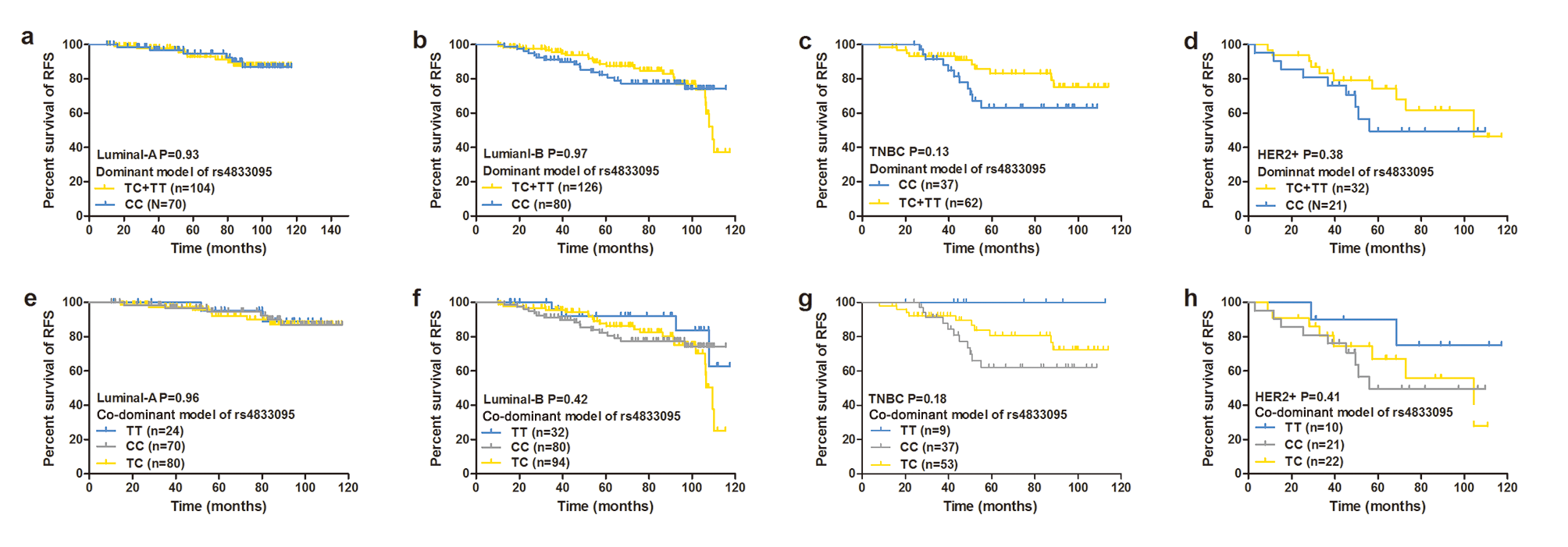

Supplement: S2 Fig — Effects of rs34833095 on RFS according to different models for Luminal-A: (a) dominant model, (b) co-dominant model; Luminal-B: (c) dominant model, (d) co-dominant model; TNBC: (e) dominant model, (f) co-dominant model; and HER2+ subtype: (g) dominant model, (h) co-dominant model. P-value tested by the log-rank test. (TIF) [file pone.0133184.s002.tif]
